# Recent Progress on the Electrochemical Biosensing of *Escherichia coli* O157:H7: Material and Methods Overview

**DOI:** 10.3390/bios10050054

**Published:** 2020-05-18

**Authors:** Nasrin Razmi, Mohammad Hasanzadeh, Magnus Willander, Omer Nur

**Affiliations:** 1 Physics and Electronics, Department of Sciences and Technology, Linköping University, SE-601 74 Norrköping, Sweden; omer.nour@liu.se; 2 Pharmaceutical Analysis Research Center, Tabriz University of Medical Sciences, Tabriz 51664, Iran; mhasanzadeh2@gmail.com

**Keywords:** *E. coli* O157:H7, electrochemical biosensors, biomedical analysis, environmental monitoring, portable biodevice, biotechnology

## Abstract

*Escherichia coli* O157:H7 (*E. coli* O157:H7) is a pathogenic strain of *Escherichia coli* which has issued as a public health threat because of fatal contamination of food and water. Therefore, accurate detection of pathogenic *E. coli* is important in environmental and food quality monitoring. In spite of their advantages and high acceptance, culture-based methods, enzyme-linked immunosorbent assays (ELISAs), polymerase chain reaction (PCR), flow cytometry, ATP bioluminescence, and solid-phase cytometry have various drawbacks, including being time-consuming, requiring trained technicians and/or specific equipment, and producing biological waste. Therefore, there is necessity for affordable, rapid, and simple approaches. Electrochemical biosensors have shown great promise for rapid food- and water-borne pathogen detection. Over the last decade, various attempts have been made to develop techniques for the rapid quantification of *E. coli* O157:H7. This review covers the importance of *E. coli* O157:H7 and recent progress (from 2015 to 2020) in the development of the sensitivity and selectivity of electrochemical sensors developed for *E. coli* O157:H7 using different nanomaterials, labels, and electrochemical transducers.

## 1. Introduction

The rapid spread of pathogenic bacteria, as well as their rapid development of antibiotic resistance, has caused worldwide concern as they are a major source of both foodborne and waterborne illnesses [1,2,3]. Pathogenic strains of bacteria are the main concern for environmental biology, hospitals, water supplies, and the food industry because of the diverse illnesses that microbial infection can cause, some of which can lead to death [4]. Contamination of food resources has led to the occurrence of certain diseases, placing a heavy responsibility on food distributors to restrict outbreaks [2,5,6]. More important, the majority of water sources are contaminated with pathogenic bacterial strains, such as *Salmonella*, *Staphylococcus*, and *Escherichia coli*, resulting in typhoid fever, gastroenteritis, cholera, and several diarrheal responses [2,7,8]. According to the 2016 report of the World Health Organization (WHO), 829,000 annual deaths from diarrhea occurred due to bacterial water contamination [9]. Around 600 million—nearly 1 in 10 people in the world—become ill due to the consumption of contaminated food, resulting in 420,000 deaths every year and the loss of 33 million healthy life years (disability adjusted life years; DALYs) [10]. *E. coli* is a fecal coliform bacterium found in the human gut and other warm blooded animals, and is typically harmless to humans [1,11]. However, pathogenic groups of *E. coli* strains can cause diarrheal illnesses. Pathogenic *E.coli* can be categorized into six groups, including diffusely adherent *E. coli* (DAEC), enterohemorrhagic *E. coli* (EHEC), enteroaggregative *E. coli* (EAEC), enteropathogenic *E. coli* (EPEC), enteroinvasive *E. coli* (EIEC), and enterotoxigenic *E. coli* (ETEC) [12,13,14,15,16]. One of the most important EHEC pathogens is *E. coli* O157:H7 due to its ability to cause bloody diarrhea, leading to potentially fatal hemolytic uremic syndrome (HUS). The O157:H7 serotype is one of the Shiga-toxin-producing *E. coli* (STEC) strains and causes worldwide infections [16]. Since its discovery in 1982, *E. coli* O157:H7 has appeared as an significant enteric, extremely infective water- and food-borne pathogen presenting a massive challenge to public health and financial stability in terms of medical cost [17]. The transmission of *E. coli* O157:H7 mostly occurs through the consumption of food, vegetables, milk, meat, and water sources that have come in contact with fecal matter at any point [2,18,19,20]. The ingestion dose of 10–100 cells of *E. coli* O157:H7 [21] can cause respiratory failure [22,23], seizures [24,25], gastrointestinal illness, renal failure, anemia [26], HUS, hemorrhagic colitis, as well as acute kidney failure and, finally, death, particularly in infants and immunocompromised individuals [1,5,8,27,28]. Therefore, a rapid, selective, sensitive, simple, accurate, and easy-to-use method for the determination and quantification of *E. coli* O157:H7 is an urgent task in the fields of environmental monitoring, clinical diagnosis, and food safety. Traditional methods for bacterial detection via standard microbiological approaches, including pre-enrichment, selective enrichment, biochemical screening, serological confirmation, and toxin testing, are time consuming (requiring 2–6 days for the result and confirmation), laborious, and vague in terms of results [20,29,30,31]. Plate culture, polymerase chain reaction (PCR), and enzyme-linked immunosorbent assay (ELISA) are currently the typically used detection methods for *E. coli* 0157:H7 [32,33]. The conventional plate culture method requires laborious procedures that require a relatively long time to get the result. Based on specific PCR variation, the detection time could take 5–25 h. Although PCR methods, mainly real time PCR, have been vastly used for *E. coli* O 157:H7 identification by targeting some virulence factor-encoding genes, it has disadvantages, including that most of the genes are not specific for this bacterium, and difficulty in differentiating between viable and nonviable cells. In addition, this method needs specific instrumentation and is time-consuming and complicated [30,33,34]. ELISA is an immunological technique which employs an enzyme for the detection of an antigen or antibody as a result of microbial presence in a sample. These techniques often require enrichment or purification steps and pretreatments, lengthening the analysis time. To overcome these drawbacks, effort has gone into the development of a rapid, sensitive, selective, and simple pathogen detection approach that provides accurate detection. For rapid detection methods, a lot of effort has focused on the improvement of reliability, specificity, feasibility in various environments, speed, cost, and miniaturization [16,35]. Recently, biosensors have become a more sensible option for the detection of *E. coli* O157:H7, as they are highly rapid, sensitive, selective, and provide accurate identification and quantification. Biosensors are defined as analytical devices using biological/biochemical reactions for detection of target analytes, and essentially consist of a bio-element and a transducer [36,37,38,39]. A biosensor should be able to give quantitative or semiquantitative information and detect the target molecule without requiring any additional processing steps. The measurement approach could be simply in a droplet format or in a continuous flow format. The performance of an ideal biosensor for pathogenic bacteria detection is summarized in Table 1 [40]. Biosensors have the advantages of simplicity, specificity, low detection limit, simple operation, being inexpensive, easy to use, providing real-time measurement, capability of multitarget testing and automation, portability, miniaturization, and rapid detection. In recent years, biosensors with different transducers have been extensively applied for pathogenic bacteria detection. Among the different types of transducers, electrochemical biosensors have gained more attention due to their simplicity and sensitivity. This review gives a general overview of the reported electrochemical methods from 2015 to 2020 for the rapid detection of *E. coli* O157:H7.

## 2. Electrochemical Biosensors for the Detection of *E. coli* O157:H7

Electrochemical biosensors are frequently designed and have been widely used for the detection of food-borne and water-borne pathogens due to the possibility of miniaturization and construction of disposable, flexible, and cheap sensing systems. As reported by the International Union of Pure and Applied Chemistry (IUPAC), an electrochemical biosensor is an independently integrated system using a bioreceptor in contact with an electrochemical transduction part that provides specific quantitative or semiquantitative analytical data [41,42]. The current produced by oxidation and reduction reactions related to the presence of the electroactive species or its rate of production/consumption is measured by an electrochemical biosensor. The produced electrical signal is proportional to the target’s concentration [43,44]. Electrochemical biosensors are categorized into four classes: impedance, amperometric, conductometric, and potentiometric, according to the nature of the electrochemical changes detected via the biorecognition reaction [38]. Simplicity and speed are the key advantages of electrochemical biosensors. Low-cost electrodes incorporated with simple electronics allow rapid detection in easy-to-use, miniaturized portable devices. For environmental monitoring, the capability to detect the target concentration within a complex sample at the point-of-care and in real time is particularly interesting [38,45]. The number of papers reported for the detection of *E. coli* O157:H7 using an electrochemical transducer from 2015 to 2020 is large. Table 2 summarizes the studies related to using an electrochemical transducer for the detection of *E. coli* O157:H7. As shown in Table 2, many of the studies are devoted to genosensors and immunosensors. Genosensors use DNA sequencing analysis for bacterial detection. Nucleic acid hybridization is based on the immobilization of a single-stranded DNA sequence on a specific substrate. An obtained electrical current signal is the result of the binding of a complementary DNA sequence to the probe DNA. Detection of a specific DNA sequence provides a rapid, simple, cost-effective, and physically small assay that can be operated by nonprofessional users [46,47]. Electrochemical immunosensors rely on an electrochemical signal resulting from stable antigen–antibody complex formation, allowing highly sensitive detection. A label or marker attached to an antibody (Ab) or an antigen (Ag) is required for labeled electrochemical immunosensors to achieve electron transfer. The detected amount of the label corresponds to the target analyte’s concentration. In sandwich-based immunosensors, two specific antibodies are used to capture the target cell. One of the antibodies is immobilized on the surface of the electrode and the other one is labeled with an electroactive marker or a label which can produce an electroactive product [48,49]. For detection of pathogenic bacteria, different kinds of nanomaterials have been integrated into the biosensors, yielding improvements in terms of stability, sensitivity, selectivity, and speed of the electrochemical biosensors. As shown in Table 2, gold nanoparticles and nanostructures have gained considerable attention for the detection of *E. coli* O157:H7. Providing a stable biomolecule immobilization while retaining their bioactivity is the major advantage of using gold nanoparticles in electrochemical biosensors. Application of gold nanomaterials in an electrochemical biosensor offers improvements in signal amplification, electron transfer, and electrocatalytic activity. The unique properties of gold nanoparticles, including their inert nature in biological fluids, biocompatibility, presence of functional groups for binding ligands, high surface to volume ratio, etc., make their use promising in the construction of electrochemical biosensors [50]. In this review, an attempt has been made to organize the recently reported studies using electrochemical transducers (Table 2) for the detection of *E. coli* O 157:H7. The current challenges and future directions are discussed.

### 2.1. Voltammetric-Based Biosensors

**Figure 1:**
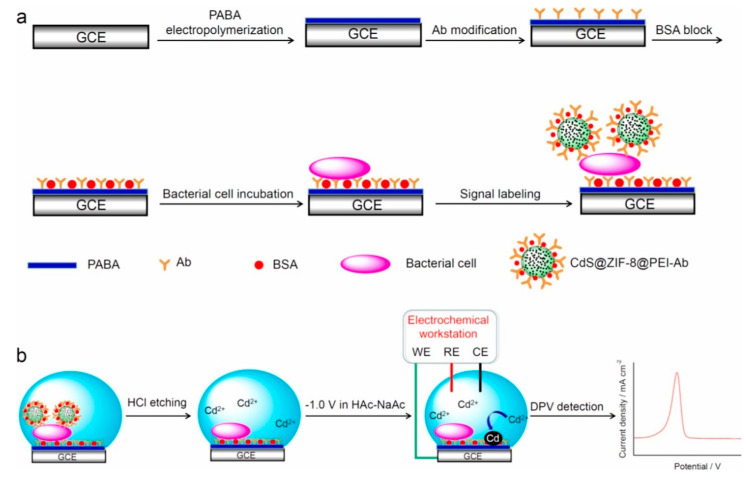
(**a**) Schematic diagram of the fabricated steps of the electrochemical biosensor for *E. coli* O157:H7 using CdS@ZIF-8 as signal tags, (**b**) Illustration of the detection steps by DPV. (GCE, glassy carbon electrode; PABA, Poly(*p*-aminobenzoic acid); Ab, antibody; BSA, bovine serum albumin; Cds, cadmium sulfide quantum dots; ZIF-8, zeolitic imidazolate framework-8; PEI, polyethyleneimine; WE, working electrode; RE, reference electrode; CE, counter electrode; HCL, hydrochloric acid; DPV, differential pulse voltammetry) [59].

**Figure 2:**
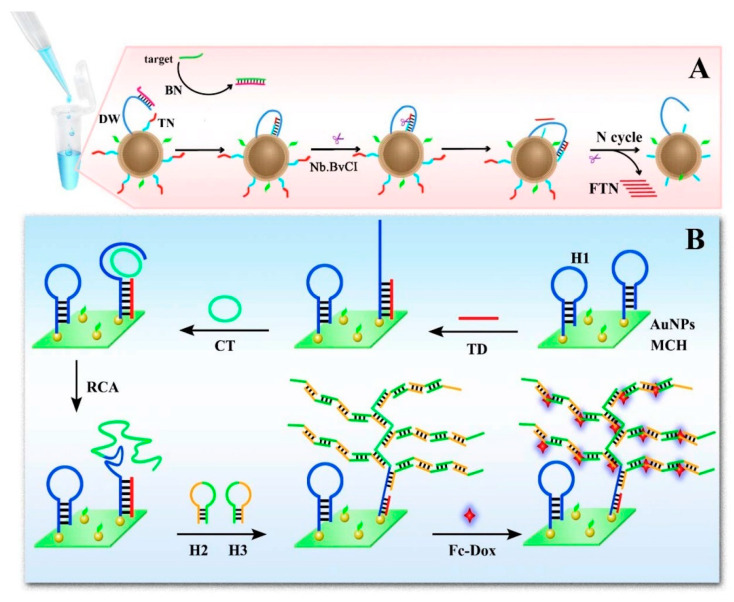
Schematic illustration of the electrochemical biosensor for *E. coli* O157:H7 detection. (**A**) Schematic illustration of the 3D DNA walker-based amplification reaction triggered by the target gene for transfer oligonucleotide fragment production; (**B**) Illustration of amplification reactions based on HCR and RCA on the surface of electrode to produce long double stranded DNA sequences for greater immobilization of electrochemical indicators associated with the target gene’s concentration. (Au NPs, gold nanoparticles; BN, blocking DNA; CT, circular template; DW, DNA walker; FTN, fragment of TN; H1, hairpin DNA1; H2, hairpin DNA2; H3, hairpin DNA3; HCR, hybridization chain reaction; MCH, 6-mercapto ethanol; RCA, rolling circle amplification; TN, transfer oligonucleotide) [60].

Voltammetric measurement is based on the principle of measuring the flowing current produced through the working electrode dipped in a solution containing an electroactive species. The easy recognition of the target via its voltammetric peak potential qualifies voltammetry as a strong electrochemical technique in biosensing [44,87]. Cyclic voltammetry (CV), square wave voltammetry (SWV), and differential pulse voltammetry (DPV) are the frequently applied techniques in voltammetric biosensors. In the past few years, numerous approaches using aptamers, enzymes, and nanomaterials have been successfully incorporated into voltammetric biosensors for the detection of *E. coli* O 157:H7. Zhong and coworkers proposed a new electrochemical biosensor for the detection of *E. coli* O157:H7 (Figure 1). As signal-amplifying tags for the determination, cadmium sulfide quantum dots (CdS QDs) and encapsulated zeolite imidazolate framework-8 (ZIF-8) particles were used. In the presence of CdS QDs, the growth of ZIF-8, CdS@ZIF-8 muticore–shell particles on the sample was achieved. In order to introduce amino groups on the surface, CdS@ZIF-8 particles were coated via polyethyleneimine, followed by an anti-*E. coli* O157:H7 antibody modification on the surface for the selective detection of *E. coli* O157:H7. CdS@ZIF-8 particles, as signal tags, were used for preparing a sandwich-based sensor. By HCl leaching, Cd (II) ions were released from CdS@ZIF-8, leading to *E. coli* O157:H7 detection by differential pulse voltammetry. The linear range of 10 to 10^8^ colony forming units (CFU)/mL and 3 CFU/mL was achieved by the fabricated immunosensor which also showed good sensitivity and selectivity of *E. coli* O157:H7 in milk samples. The proposed biosensor can be expanded to be used for detection of other pathogenic bacteria [59]. Very recently, Yan li and coworkers [60] used multiple amplification strategies via 3D DNA walker, rolling circle amplification (RCA), and hybridization chain reaction (HCR) to develop a sensitive and selective electrochemical biosensor for the accurate determination of *E. coli* O157:H7 (Figure 2). The target sequence of the *E. coli* O157:H7 was extracted, transformed, and amplified. After that, in order to generate an enhanced electrochemical signal, a large sequence of double-stranded DNA as a result of HCR progress, immobilized electrochemical indicators. Based on the proposed strategy the detection limit was 7 CFU/mL for *E. coli* O157:H7 with a linear range of 10 to 10 × 10^4^ CFU/mL. The proposed multiple amplification strategy-based biosensor can be readily used for determination of different microorganisms, allowing a novel approach for early diagnosis of malignancies and monitoring the therapy responses [60]. The advantage of the voltammetric technique is that it provides highly sensitive measurements and the possibility of simultaneous detection of multiple analytes. This technique can provide low LODs of 2 CFU/mL using an electrochemical aptasensor detection strategy based on amino-functionalized metal-organic frameworks. Despite the low LOD achievements in some of the studies, testing the fabricated biosensors in real and complex samples remains to be done. Moreover, the detection time in most of the conducted studies was long, which needs to be improved. Research towards the simultaneous detection of *E. coli* O157:H7 in complex and real samples is also a major requirement.

### 2.2. Impedimetric Based Biosensors

**Figure 3:**
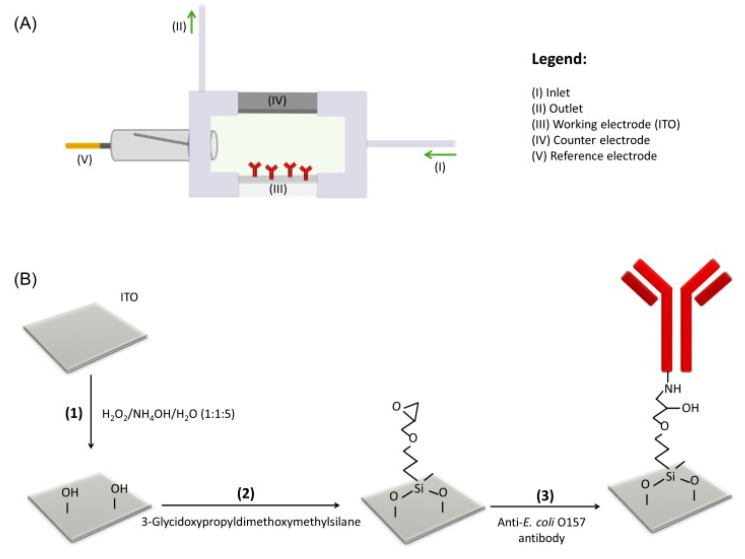
Schematic illustration of electrochemical cell (**A**) fabrication of immunosensor (**B**): hydroxylation (1), silanization (2), and antibody binding (3) [62].

**Figure 4:**
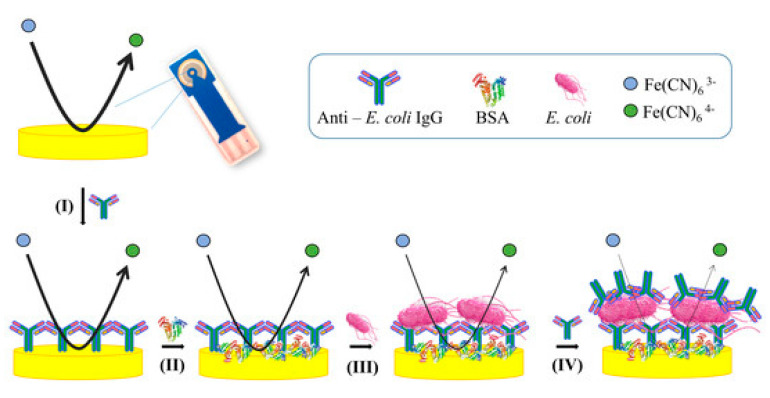
Schematic illustration of the stepwise functionalization and detection of the proposed immunosensor. The black line shows the redox reaction intensity, which is inhibited as the surface covering grows. Its thickness reduction is related to decrease of the “effective” area available for the electrolyte current, which is measured through an increase of the charge transfer resistance. (I: functionalization of the surface with antibodies, II: blocking the free remaining spaces on the gold electrode by BSA, III: reaction of immobilized antibodies and the *E. coli* cells, IV: conveying a fresh anti-E. coli Abs solution to the circuit) [73].

Impedimetric biosensors are one of the earliest approaches developed for rapid pathogen detection [88]. The main difference between this technique and other electrochemical techniques is conductivity detection [38]. Impedimetric biosensors work by analyzing the electron transfer at the electrode surface or measuring the solution/medium conductivity, which can be read as an impedance response [38]. The most frequently used technique for impedimetric biosensors is electrochemical impedance spectroscopy (EIS). This technique scans the detection volume and uses an electrical frequency sweep in the range of 10 KHz to 10 MHz [35,38]. Electrochemical impedance spectroscopy (EIS) is an easily operated, simple, straightforward, and sensitive technique which has attracted substantial interest for *E. coli* O157:H7 determination. Barreiros de Santos and colleagues developed an indium tin oxide (ITO)-based impedimetric biosensor by using a robust, simple, and direct approach for the detection of *E. coli* O157:H7 (Figure 3). Immobilization of anti-*E. coli* antibodies onto ITO electrodes was done, and epoxy silane on the surface of ITO was attached covalently, as shown by atomic force microscopy and cyclic voltammetry. By using optical waveguide light mode spectroscopy (OWLS), antibody immobilization on the epoxy silane layer was quantified and a mass variation of 12 ng cm^−2^ (0.08 pmol cm^−2^) was achieved. The selectivity of the antibodies and functionalization procedure’s efficiency were confirmed by achieving a ratio of 1:500 *Salmonella typhimurium*/*E. coli* O157:H7. The proposed ITO-based immunosensor was evaluated by electrochemical impedance spectroscopy. A very low limit of detection of 1 CFU mL^−1^ with a large linear working range of 10–10^6^ CFU mL^−1^ was achieved by using electrochemical impedance spectroscopy. The 20% detection of nonspecific bacteria, made up of *E. coli* K12 and *S. typhimurium,* showed the specificity of the impedimetric immunosensor, meaning that ITO is highly selective and sensitive [62]. Lan Yao and coworkers developed a microfluidic impedance biosensor for sensitive, rapid, and continuous *E. coli* O157:H7 detection by applying immune magnetic nanoparticles. For biological signal amplification, urease was used. In order to make the immune magnetic nanoparticles (MNPs), streptavidin-modified MNPs conjugated with biotinylated polyclonal antibodies were used. To make the MNP–bacteria complexes, the target is separated by the MNPs. Afterwards, to form the MNP–bacteria–gold nanoparticles (GNP)–urease complex, the gold nanoparticles modified with the urease and aptamers with the MNP-bacteria were conjugated. Then, hydrolysis of urea into ammonium carbonate led to impedance decrease. A low detection limit of 12 CFU/mL was obtained by online impedance measurement [64]. Recently, Martina Cimafonte and coworkers developed an electrochemical impedance immunosensor based on a screen-printed gold electrode by immobilizing anti-*E. coli* antibodies onto the gold surface covalently by the photochemical immobilization strategy for fast *E. coli* determination in water (Figure 4). In this study, in order to develop an “on-off” electrochemical impedimetric immunosensor, photochemical immobilization technique (PIT) was used for the first time in the functionalization of commercial gold electrodes using Fe (CN)_6_^3−^/Fe (CN)_6_^4−^ as a redox probe. *E. coli* in drinking water was selectively and sensitively detected with a limit of detection of 3 × 10 CFU/mL. The proposed biosensor needed no preconcentration or pre-enrichment steps for the detection process [73]. The ability to perform label-free detection is the most important advantage of impedimetric electrochemical biosensors; however, in some of the conducted studies, labels have been used for signal amplification. As shown in Table 2, by using an impedimetric sensing strategy, label-free direct detection of *E. coli* O157:H7 with a LOD of 1 CFU/mL was achieved. However, substantial time was required for the patterning of anti-*E. coli* O157 antibodies. Moreover, a low limit of quantification and testing of the fabricated biosensors in real and complex samples in most of the studies have not been achieved.

### 2.3. Amperometric-Based Biosensors

**Figure 5:**
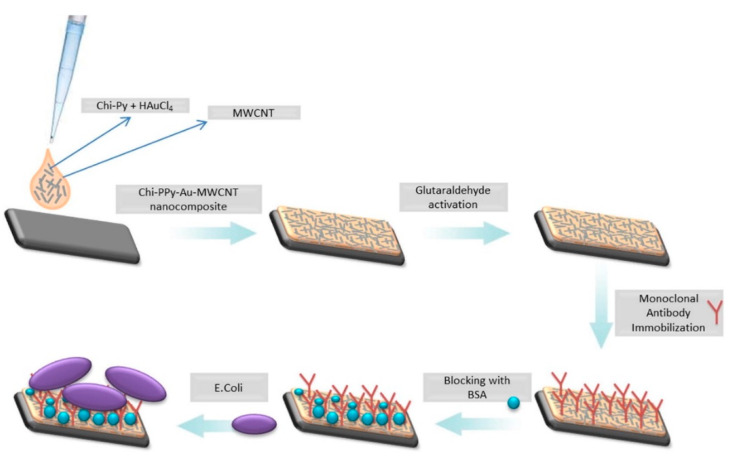
Schematic illustration of the experimental setup of the immunosensor [77].

**Figure 6:**
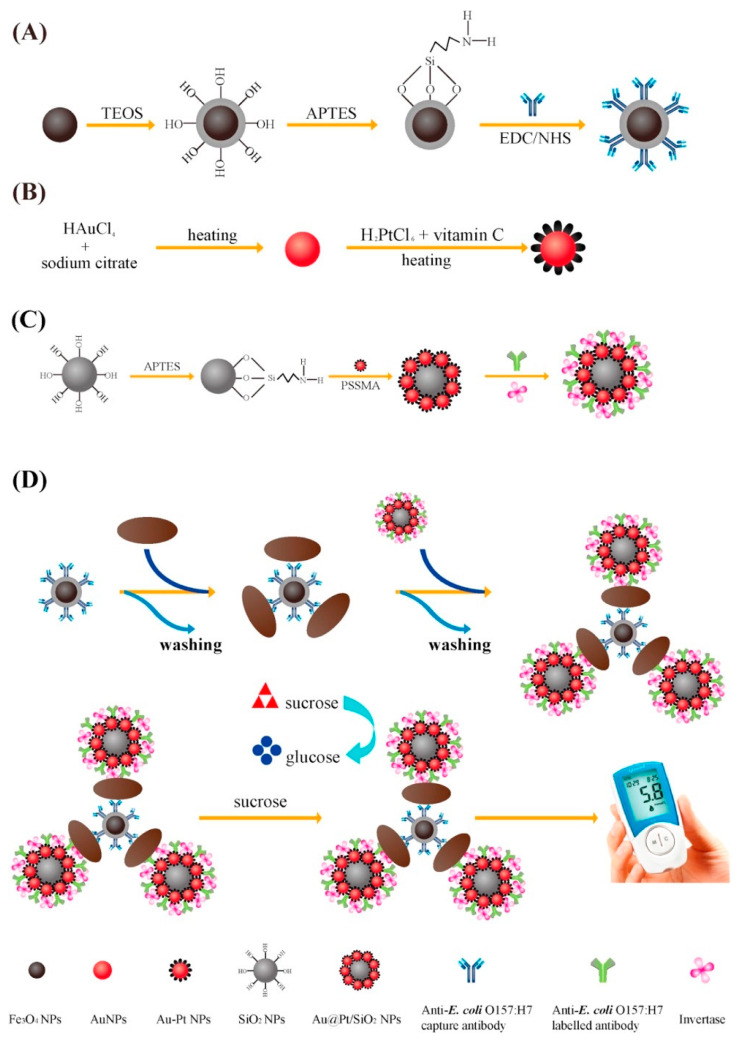
A schematic diagram of the preparation process of Ab-Fe_3_O_4_@SiO_2_ NPs (**A**), Au@Pt NPs (**B**), Ab/invertase-Au@Pt/SiO_2_ NPs (**C**); Experimental process of *E. coli* O157:H7 detection employing Ab-Fe_3_O_4_@SiO_2_ NPs and Ab/invertase-Au@Pt/SiO_2_ NPs based on PGM (**D**) [76].

As a class of electrochemical biosensors, amperometric biosensors transduce the biological recognition reactions caused by electroactive agents at the electrode surface into a current signal to determine the target molecule within a sample matrix. They can be integrated with nucleic acids, enzymes, and antibody recognition elements, and are applicable for environmental monitoring [38,44]. Differential pulse voltammetry, cyclic voltammetry, and square wave voltammetry are different amperometric methods which are applied in biosensors. The false current reading because of the electroactive interference present in the sample matrix is the limitation of this technique, and can be solved by various methods such as changing the analyte, diluting the sample, etc. [38,44]. Ahmet Guner and coworkers developed a highly sensitive sandwich assay electrochemical immunosensor based on a Py, Pyrrole/gold nanoparticles/multiwalled carbon nanotube/chitosan (PPy/AuNP/MWCNT/Chi) hybrid nanobiocomposite-modified pencil graphite electrode (PGE) for *E. coli* O157:H7 detection (Figure 5). The hybrid bionanocomposite platform was modified with anti-*E. coli* O157:H7 monoclonal antibodies and the product was characterized by using cyclic voltammetry. A detection limit of 30 CFU/mL in PBS buffer with a linear range of 3 × 10 to 3 × 10^7^ CFU/mL was achieved. For application in food quality and safety control, the produced sensor showed high stability and reproducibility [77]. In another study, Lingxian Ye et al. proposed a sensitive point-of-care testing (POCT) with Au-Pt bimetallic nanoparticle (Au@Pt)-functionalized silica nanoparticles (SiO_2_ NPs) and Fe_3_O_4_ magnetic nanoparticles (Fe_3_O_4_ NPs) for *E. coli* O157:H7 determination (Figure 6). As a negatively charged polyelectrolyte, poly-(4-styrenesulfonic acid-co-maleic acid) (PSSMA) coated on the amino group modified the SiO_2_ NPs surface, conferring electrostatic force. The PSSMA applied to connect the negatively charged Au@Pt NPs to the SiO_2_ NPs led to the formation of Au@Pt/SiO_2_ NPs. As signal labels, antibody- and invertase-conjugated Au@Pt/SiO_2_ NPs were used. In order to enrich and capture the target in a positive sample, monoclonal antibody-functionalized magnetic nanoparticles (Ab-Fe_3_O_4_@SiO_2_ NPs) were used. For the quantitative readout by the PGM, the invertase in the proposed sandwich assay catalyzed the hydrolysis of sucrose to generate a large amount of glucose. A low detection limit of 1.83 × 10^2^ CFU/mL was achieved [76]. Electrochemical biosensors based on amperometry have the advantages of high sensitivity, rapid, low cost, and robustness with the possibility of portability. Focusing of these aspects, more studies need to be performed in order to provide a portable sensitive biosensor for the detection of *E. coli* O157. Multiplexing detection and assessing the reproducibility are other parameters which should be focused on while conducting future studies based on amperometry. As the limit of quantification is rarely calculated in the reviewed studies, it also should be included to enable comparison between the reported studies.

### 2.4. Potentiometric-Based Portable Baiosensors

**Figure 7:**
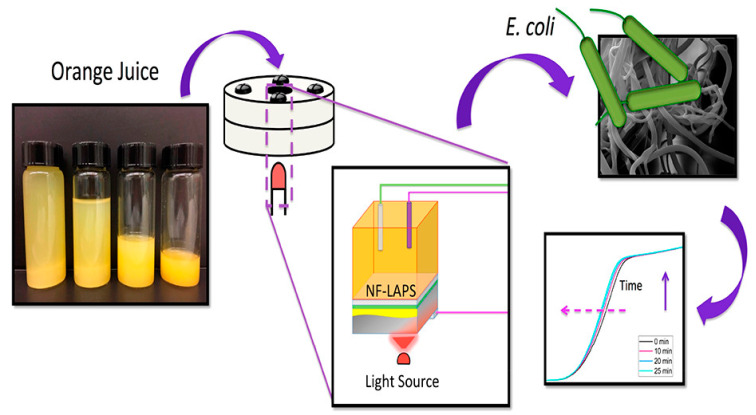
Schematic illustration of the nanofiber-light addressable potentiometric sensor (NF-LAPS) sensor comprising a three-electrode system [86].

Recently, efforts have been focused on the design of portable sensors for low-cost, on-site, and fast *E. coli* O157:H7 detection due to the zero-tolerance policy concerning its presence in food. The portability of electrochemical biosensors is of critical importance to realize in-field determination of foodborne and waterborne microorganisms [35]. Few electrochemical lab-on-a-chip and portable biosensors have been made for *E. coli* determination. Potentiometric biosensors, as low-cost, small, and highly sensitive and selective sensors, apply an ion-selective electrode and ion-sensitive field effect transistor to acquire analytical data [38]. Recently, Parmiss Mojir Shaibani and coworkers (Figure 7) reported a paper for the detection of *E. coli* in orange juice using a portable nanofiber-light addressable potentiometric sensor (NF-LAPS). As the sensitive layer, electrospun pH-sensitive poly (vinyl alcohol)/poly(acrylic acid) (PVA/PAA) hydrogel NFs was chosen. A limit of detection of 100 CFU/mL was obtained selectively in less than one hour by using NF-LAPS [86].

### 2.5. Nanoimpact Method

During the last decade, a powerful new electrochemical technique named the “nanoimpact method” has been developed for single bacterial cell characterization and detection. The method is based on the Faradaic charge transfer following particle collision. Diffusional Brownian motion causes particle movement, and due to the interaction of suspended particles with the electrode, under an oxidizing potential, a short current burst results from the interaction between the particles and the electrode [89]. The detection of the nanoimpact is performed using the change in diffusion current. Lee et al. [90] has reported a fast electrochemical label-free approach through current blockages caused by collision events for *E. coli* single cell detection on an ultramicroelectrode. The ferrocyanide–ferricyanide redox couple was used in this study. This methodology has the capability to be used to study other pathogenic bacteria and different target molecules [90]. The problem with the surface blockage detection strategy is the lack of selectivity between various bacterial species and dead and alive cells. In another study, Couto and coworkers [91] applied a carbon microelectrode for fast redox mediated detection of *E. coli* using impact electrochemistry. N,N,N′,N′-tetramethyl-1,4-phenylenediamine (TMPD) was used as a redox mediator to interact with cytochrome c oxidases of bacteria and obtain an electrochemical current, or “on” signals. The advantage of this system is the minimization of false-positive signals. The integration of the reported study with microfluidic devices may lead to the low concentration detection of bacteria [91].

## 3. Conclusions and Future Perspectives

Bacterial detection is very important in water monitoring, environmental assessment, and the food industry. *E. coli* O157:H7 has caused several outbreaks worldwide since its first recognition. Thus, the detection of *E. coli* O157:H7 is one of the major challenges to prevent severe outbreaks. Although currently available methods are highly sensitive, they require a long time to perform and are labor-intensive; therefore, there is a demand for a rapid, simple, sensitive, and low-cost alternative. Among the different types of transducers, electrochemical biosensors, owing to their fast response, selectivity, low cost, sensitivity, possibility of miniaturization, and capability of being integrated into one device, are extensively studied and well developed for the detection of *Escherichia coli* O157:H7. In this review, the recent developments of label and label-free electrochemical biosensors for the detection of *Escherichia coli* O157:H7 have been summarized. Although label-free methodologies have the advantages of direct and simple detection with a relatively low detection time and possibility of integration into one test chip, the lack of additional signal amplification and the incubation of the target bacteria with the electrodes are the main disadvantages. To overcome the interference of nontarget molecules in label-free biosensors, appropriate selection of more specific bioreceptors, such as aptamers, etc., is necessary. As illustrated in Table 2, most of the recent chemical recognition approaches involve affinity sensing strategies using aptamers and antibodies as bioreceptors. Aptamers with similar affinity and specificity are chemically stable, small, and they have a simple development process, high target potential, and less production time and cost compared to antibodies. Despite some advantages that aptamers over antibodies, some of the recent studies overviewed in this manuscript are immunosensors based on immobilized antibodies using different kinds of linkages, such as gold nanoparticles. This may be because antibodies are an established technology in all labs, whereas aptamer commercialization has not occurred as quickly as expected. However, the appropriate orientation of antibodies is the most important factor for the improvement of the performance of antibody-based immunosensors in terms of specificity and sensitivity, and the appropriate selection of nanomaterials could overcome this problem. Nanotechnology is an emerging field in science and different nanomaterials, especially gold nanoparticles, have been integrated in the development of electrochemical biosensors for *E. coli* O157:H7 detection. Nanomaterials are highly important for the immobilization of bioaffinity agents for label-free strategies, and further research should be conducted on the improvement of novel nano-scale materials for effective electron transduction. Current progress in nanotechnology is growing; further studies regarding nanomaterial stability and toxicity in aqueous environments and further progress of smart nanomaterials with useful functions with low cost are expected to solve the improve the sensitivity of electrochemical biosensors towards *E. coli* O157:H7 detection. Because of the relatively large size of whole bacteria compared to typical biological targets and the existence of different epitopes on the surface of bacteria that can lead to nonspecificity of the approach, a product for real sample applications and the commercial market is yet to be successfully developed. Although impact electrochemistry, as a promising and sensitive technique, has gained attention for *E. coli* bacteria sensing at the single cell scale, selectivity is the main challenge of this technique. Digital microfluidics, as portable and stable platforms with the power of automation, have the capability to overcome the limitations of current analytical methods in real-time applications, but remain challenging. Overall, sensitivity, specificity, stability, detection time, sample processing, size, ability to perform in different conditions, and no special training requirement are the key features of a biosensor. In addressing all these issues, electrochemical biosensors have a long way to go, but collaboration between academia and industry can pave the way for developing a desirable, portable product.

## Figures and Tables

**Table 1:** Summary of the requirements for a bacterial biosensor.

Feature	Requirement
Sensitivity	A biosensor should have the ability to detect the pathogen at very low infective dosage
Specificity	A biosensor should be able to discriminate between the target molecule and nontarget molecules
Robustness (durability)	A biosensor should have the ability to withstand different conditions, such as changes in temperature, etc.
Detection time	Analysis time should be minimal for real-time response
Reproducibility	The result should be reproducible over the period of time without failure
Ease of use	The biosensor should not require specific operator skills
Accuracy	A biosensor should not have should not have false-negative or false-positive results
Cost-effectiveness	The biosensor should be inexpensive

**Table 2:** Electrochemical sensors for *E. coli* O157:H7 detection.

Method	Assay Strategy	Material Type	Technique	LOD	Linear Range	Ref.
Voltammetric	Immunosensor	Au NPs	SWV	10 CFU/mL	10–10^6^ CFU/mL	[51]
Voltammetric	Aptasensor	-	DPV	80 CFU/mL	5 × 10^2^–5 × 10^7^ CFU/mL	[34]
Voltammetric	Aptasensor	Single wall carbon nanotube	CV–DPV	1.7 × 10 CFU/mL	1.7 × 10–1.1 × 10^7^ CFU/mL	[52]
Voltammetric	Immunoassay	SG-PEDOT-Au NPs	DPV	3.4 × 10 CFU/mL	7.8 × 10–7.8 × 10^6^ CFU/mL	[53]
Voltammetric	Genosensor	Graphene oxide-nickel ferrite-chitosan (GO/NiF/ch) film	DPV	1 × 10^−16^ M	10^−6^–10^−16^ M	[54]
Voltammetric	Bare Indium Tin Oxide (ITO) based Immunosensor	Au NPs	DPV	330 cells/mL	1–10^6^ cells/mL	[55]
Voltammetric	Aptasensor	Cu-MOF/PANIAg NPs	DPV–EIS–CV	2 CFU/mL	2.1 × 10^1^–2.1 × 10^7^ CFU/mL	[56]
Voltammetric	Dual signal amplification strategy based on double DNA hybridization	Polyaniline film and Au NPs	CV	4 CFU/mL	4 × 10^6^–4 CFU/mL	[57]
Voltammetric	Immunosensor	Reduced graphene oxide (rGO)	LSV–EIS	4 CFU/mL	4 × 10^8^–4 CFU/mL	[58]
Voltammetric	Sandwich type immunosensor	Cadmium Sulfide quantum dots in zeolitic imidazolate framework (CdS@ZIF-8) nanoparticles	DPV	3 CFU/mL	10–10^8^ CFU/mL	[59]
Voltammetric	Multiple amplification strategy via 3D DNA walker	AU NPs	CV–EIS–DPV	7 CFU/mL	10–10^4^ CFU/mL	[60]
Impedimetric	Interdigitated label free microelectrode	-	EIS	7 cells/mL	7.2 × 10^0^–7.2 × 10^8^ cells/mL	[6]
Impedimetric	Immunosensor	Streptavidin coated magnetic beads (MBs)	EIS	10^3^ CFU/mL	10^2^–10^6^ CFU/mL	[61]
Impedimetric	Label free ITO based immunosensor	-	EIS	1 CFU/mL	10–10^6^ CFU/mL	[62]
Impedimetric	Lectin functionalized mixed self-assembled monolayer	11- mercaptoundecanoic acid (MUA) and dithiothreitol (DTT)	EIS–CV	75 cells/mL	1 × 10^2^–1 × 10^5^ cells/mL	[29]
Impedimetric	Immunosensor	Graphene wrapped copper (II) assisted cysteine hierarchical structure	EIS	3.8 CFU/mL	10–10^8^ CFU/mL	[63]
Impedimetric	Aptasensor based on Urease catalysis amplification strategy	Streptavidin modified magnetic nanoparticles, Gold NPs	EIS	12 CFU/mL	10–10^5^ CFU/mL	[64]
Impedimetric	self-assembled monolayer based immunoassay	-	EIS	1 × 10^2^ CFU/mL	10^2^–10^7^ CFU/mL	[65]
Impedimetric	Ab based magneto impedance sensor	Gold nanofilm	-	50 CFU/mL	50–500 CFU/mL	[66]
Impedimetric	Multiple interdigitated electrode array	Gold thin film	IS	39 CFU/mL	-	[67]
Impedimetric	Microelectromechanical system (MEMS) biosensor based on Ab	Gold thin film	IS	13 CFU/ML	-	[68]
Impedimetric	Immunosensor	Magnetic nanobeads	–	10^4.45^ CFU/mL	10^4^–10^7^ CFU/mL	[69]
Impedimetric	Immunosensor	Cu_3_(BTC)_2_/PANI	EIS	2 CFU/mL	2-2 × 10^8^ CFU/mL	[70]
Impedimetric	Aptasensor	streptavidin modified MNPs, Au NPs	EIS	10 CFU/mL	10–10^4^ CFU/mL	[71]
Impedimetric	Immunosensor	Au NPs	IS	100 CFU/mL	300–10^5^ CFU/mL	[26]
Impedimetric	DNA sensor	3-Aminipropyl trimethoxysilane (APTES) and GA	EIS	0.5–25 pg/10 mL	0.1 pg/10 mL	[72]
Impedimetric	Immunosensor	Gold print	EIS	3 × 10 CFU/mL	10–10^8^ CFU/mL	[73]
Impedimetric	DNA biosensor	Graphene oxide Chitosan Hybrid nanocomposite	CV–EIS	3.584 × 10^−15^ M	1 × 10^−14^–1 × 10^−8^ M	[74]
Amperometric	Hydrogen evolution reaction based immunosensor	Au NPs	CV–CA	309 CFU/mL	10^2^–10^5^ CFU/mL	[75]
Amperometric	Personal Glucometer (PGM) Immunoassay	Au@Pt/SiO_2_ NPsand Fe_3_O_4_@SiO_2_ NPs	-	1.83 × 10^2^ CFU/mL	3.5 × 10^2^–3.5 × 10^8^ CFU/mL	[76]
Amperometric	Immunosensor	PPy/AuNP/MWCNT/Chi bionanocomposite	CV	30 CFU/mL	3 × 10–3 × 10^7^ CFU/mL	[77]
Amperometric	DNA biosensor	GOx–Thi–Au@SiO_2_ nanocomposites	CV–DPV	0.01 nM	0.02–50 nM/L	[78]
Amperometric	Nonenzymatic immunoassay	Silica coated Fe_3_O_4_ magnetic nanoparticles and Au@Pt nanoparticles	CV	4.5 × 10^2^ CFU/mL	4 × 10^3^–4 × 10^8^ CFU/mL	[79]
Amperometric	Genosensor	Cd NPs	CV–EIS–DPV	1.97 × 10^−14^ M	1.94 × 10^–13^ and 2.01 × 10^–14^ M	[80]
Amperometric	Screen printed interdigitated electrode	core–shell magnetic beads and Au NPs	CV	52 CFU/mL	10^2^–10^6^ CFU/mL	[81]
Amperometric	DNA based sensor	3-aminipropyl triethoxysilane (APTES)	CA	0.8 fM	1 fM–10 µM	[82]
Amperometric	Immunosensor	MNPs and Au NPs	DPV	10 CFU/mL	10^1^–10^6^ CFU/mL	[83]
Amperometric	Genosensor	Carboxylated graphene nanoflakes (Cx-Gnfs)	CV–EIS–CA	10^−17^ M	10^−6^–10^−17^ M	[84]
Amperometric	Genosensor	Reduced graphene oxide (rGO)	CV–EIS–CA	10^−15^ M	10^−6^–10^−17^ M	[84]
Amperometric	Aptasensor	Au NPs	CV	10 CFU/mL	10–10^9^ CFU/mL	[85]
Potentiometric	pH sensitive nanofibre	poly(vinyl alcohol)/poly(acrylic acid) (PVA/PAA) hydrogel NFs	–	10^2^ CFU/mL	10^2^–10^6^ CFU/mL	[86]

Abbreviations: CV: Cyclic voltammetry, SWV: Square wave voltammetry, DPV: Differential pulse voltammetry, EIS: Electrochemical impedance spectroscopy, IS: Impedance spectroscopy, CA: Chronoamperometry, LSV: Linear sweep voltammetry.
